# Complete Genome Sequences of Seven *Peanut Stunt Virus* Strains from Japan

**DOI:** 10.1128/MRA.00952-18

**Published:** 2018-09-27

**Authors:** Hiroyuki Takahashi, Ami Ogawa, Sota Inoue, Ryosuke Yasaka, Kazusato Ohshima, Masashi Ugaki, Masashi Suzuki

**Affiliations:** a Laboratory of Bioresource and Technology, Department of Integrated Biosciences, Graduate School of Frontier Sciences, The University of Tokyo, Kahiwa, Chiba, Japan; b Laboratory of Plant Virology, Department of Applied Biological Sciences, Faculty of Agriculture, Saga University, Honjo-machi, Saga, Japan; c The United Graduate School of Agricultural Sciences, Kagoshima University, Kagoshima, Japan; Georgia Institute of Technology

## Abstract

We present the complete genomic RNA sequences of seven isolates of *Peanut stunt virus* discovered in diseased legume plants in various regions in Japan. These sequences and the published viral sequences were compared with respect to nucleotide percentages. Their phylogenetic analysis showed that all the isolates belong to subgroup IA.

## Abstract (precis)

We present the complete genomic RNA sequences of seven isolates of *Peanut stunt virus* discovered in diseased legume plants in various regions in Japan. These sequences and the published viral sequences were compared with respect to nucleotide percentages.

## ANNOUNCEMENT

*Peanut stunt virus* (PSV) is a member of the genus *Cucumovirus* in the family *Bromoviridae* and has tripartite positive-sense single-stranded genomic RNAs 1, 2, and 3, which are 3,300, 2,900, and 2,200 nucleotides long, respectively. PSV is an economically important pathogen of legumes worldwide. RNAs 1 and 2 encode the 1a and 2a proteins, respectively, which are necessary for virus replication. RNA 2 also encodes the 2b protein, which is synthesized from a subgenomic RNA, 4A. The 2b protein is a suppressor of RNA silencing and is required for long-distance movement (1). RNA 3 encodes the 3a protein and the coat protein (CP), which is expressed from a subgenomic RNA, 4. The PSV isolates are currently classified into subgroups I to IV (2–4), and it was recently proposed that subgroup I be divided into IA, IB, and IC (5).

Previously, a Japanese strain of PSV, PSV-J, was described by Tsuchizaki (6), and the complete nucleotide sequences of its genomic RNAs were determined by Karasawa et al. (7, 8). We sequenced and constructed full-length cDNA clones of genomes of another Japanese isolate, PSV-J2 (1, 9). In addition, some other PSVs have been isolated in the following prefectures in Japan: from soybean in Kyoto (PSV-PK), Tottori (PSV-PT), and Yamagata (PSV-Y11 and PSV-Y62); from peanut in Chiba (PSV-P1) and Mie (PSV-PnT); and from pea in Gifu (PSV-GF). We obtained these seven viruses from the Genetic Resources Center, National Agriculture and Food Research Organization, Japan. We report here the complete nucleotide sequences of these PSVs.

PSVs were propagated in *Nicotiana benthamiana* after being isolated by single-lesion isolation using *Chenopodium quinoa* or cowpea. The 5ʹ and 3ʹ terminal regions were determined using 5ʹ rapid amplification of cDNA ends (RACE) and 3ʹ RACE systems (Invitrogen). Viral first-strand cDNA was synthesized using SuperScript III reverse transcriptase (Invitrogen) as previously described (1). Synthesized cDNA was PCR amplified by PSV-specific primer pairs with restriction sites for cloning using KOD Plus v2 DNA polymerase (Toyobo). The reverse transcription-PCR products were cloned into the pUC18 plasmid. DNA sequencing was performed by primer walking in both directions using the BigDye Terminator v3.1 cycle sequencing ready reaction kit and an Applied Biosystems 3500 series genetic analyzer. Nucleotide sequences of at least six independent clones for each genomic RNA of the PSV isolates were determined and analyzed.

The nucleotide identities between the seven PSVs and the other members of PSV (ER; subgroup IA, P; subgroup IB, Ag; subgroup IC, W; subgroup II, Mi; subgroup III, and Rp; subgroup IV) are listed in Table 1. Phylogenetic trees of PSV RNAs 1, 2, and 3 were generated with MEGA7 software using the neighbor-joining methods. The seven isolates clustered in subgroup IA with statistical significance for tree branching were assessed for 100%, 99%, and 79% of RNAs 1, 2, and 3, respectively, by performing 1,000 bootstrap replications. These results indicated that the seven isolates and the other Japanese strains, J and J2, belong to subgroup IA.

**TABLE 1:** Full-length RNA sequence comparison of PSV strains and accession numbers

Isolate	Basis of comparison	Length (nucleotides)	Nucleotide identity (%) for PSV strain (subgroup):						Accession no.
			ER (IA)	P (IB)	Ag (IC)	W (II)	Mi (III)	Rp (IV)	
P1	RNA 1	3,355	94.01	87.7	87.61	79.6	79.57	79.45	LC380678
	RNA 2	2,945	94.03	84.68	84.72	75.21	75.02	75.24	LC380679
	RNA 3	2,185	95.39	87.93	88.3	79.81	77.72	84.06	LC380680
GF	RNA 1	3,355	93.98	87.76	87.76	79.84	79.69	79.59	LC380681
	RNA 2	2,942	93.25	83.77	83.67	75.51	74.88	75.58	LC380682
	RNA 3	2,185	95.34	88.12	88.35	79.95	77.94	84.33	LC380683
PK	RNA 1	3,354	93.35	87.94	87.52	79.61	78.92	79.59	LC380684
	RNA 2	2,946	93.73	84.52	84.65	74.98	74.76	75.08	LC380685
	RNA 3	2,188	90.53	87.3	88.12	79.44	77.57	83.7	LC380686
PT	RNA 1	3,354	97.29	88.48	88.06	79.29	79.11	79.48	LC380687
	RNA 2	2,943	95.73	84.55	84.52	76.2	74.88	75.82	LC380688
	RNA 3	2,183	94.7	88.23	87.73	79.67	77.05	83.76	LC380689
PnT	RNA 1	3,356	93.78	87.91	87.41	79.61	78.9	79.54	LC380690
	RNA 2	2,946	93.76	84.55	84.72	75.08	74.79	75.08	LC380691
	RNA 3	2,188	90.49	87.3	88.12	79.37	77.62	83.79	LC380692
Y11	RNA 1	3,356	91.04	87.94	87.25	79.32	79.45	79.75	LC380693
	RNA 2	2,948	94.34	84.25	83.64	75.46	74.43	75.03	LC380694
	RNA 3	2,187	90.48	87.3	88.39	79.66	77.75	84.02	LC380695
Y62	RNA 1	3,359	90.99	87.75	87.09	79.4	79.33	79.73	LC380696
	RNA 2	2,944	94.37	84.3	83.65	75.67	74.62	75.14	LC380697
	RNA 3	2,185	90.44	87.43	88.13	80.2	77.88	84.36	LC380698

### Data availability.

The GenBank accession numbers for these viruses are listed in Table 1.
